# Tetra(chlorido/iodido)(1,10-phenanthroline)platinum(IV) hemi[di(chlorine/iodine)]

**DOI:** 10.1107/S1600536809007703

**Published:** 2009-03-11

**Authors:** Nam-Ho Kim, Kwang Ha

**Affiliations:** a School of Applied Chemical Engineering, The Research Institute of Catalysis, Chonnam National University, Gwangju 500-757, Republic of Korea

## Abstract

The asymmetric unit of the title compound, [PtCl_3.66_I_0.34_(C_12_H_8_N_2_)]·0.5(Cl_0.13_I_1.87_), contains a neutral Pt^IV^ complex and one half of a halogen molecule. The Pt^IV^ ion is six-coordinated in a distorted octa­hedral environment by two N atoms of the 1,10-phenanthroline ligand and Cl or I atoms. The refinement of the structure and the EDX analysis indicate that the compound is a solid solution in which there is some substitution of Cl for I and *vice versa*. The chemical formula of the pure state of the compound would have been [PtCl_4_(C_12_H_8_N_2_)]·0.5I_2_. In the analysed crystal, two Cl atoms are partially (*ca* 25% and 9%) replaced by I atoms, and the I_2_ mol­ecule has a minor component modelled as ICl. As a result of the disorder, the different *trans* effects of the N and Cl/I atoms are not distinct. The complex displays inter­molecular π–π inter­actions between the six-membered rings, with a centroid–centroid distance of 3.771 (4) Å. There are also weak intra­molecular C—H⋯Cl hydrogen bonds.

## Related literature

For details of some other Pt–phenanthroline complexes, see: Buse *et al.* (1977 ▶); Fanizzi *et al.* (1996 ▶); Kim *et al.* (2009*a* ▶,*b* ▶). For related Pt–bipyridine complexes, see: Hambley (1986 ▶); Hojjat Kashani *et al.* (2008 ▶). For bond-length data, see: Orpen *et al.* (1989 ▶).
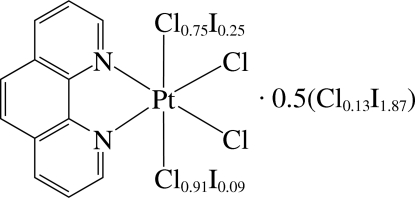


## Experimental

### 

#### Crystal data

[PtCl_3.66_I_0.34_(C_12_H_8_N_2_)]·0.5(Cl_0.13_I_1.87_)
*M* *_r_* = 669.26
Orthorhombic, 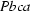

*a* = 14.215 (5) Å
*b* = 12.733 (5) Å
*c* = 17.575 (6) Å
*V* = 3180.8 (19) Å^3^
*Z* = 8
Mo *K*α radiation
μ = 11.92 mm^−1^
*T* = 293 K
0.25 × 0.17 × 0.15 mm

#### Data collection

Bruker SMART 1000 CCD diffractometer
Absorption correction: multi-scan (*SADABS*; Bruker, 2000 ▶) *T*_min_ = 0.111, *T*_max_ = 0.168
17485 measured reflections
3246 independent reflections
2169 reflections with *I* > 2σ(*I*)
*R*_int_ = 0.049

#### Refinement

*R*[*F* ^2^ > 2σ(*F* ^2^)] = 0.031
*wR*(*F* ^2^) = 0.062
*S* = 0.86
3246 reflections
194 parameters
8 restraints
H-atom parameters constrained
Δρ_max_ = 1.29 e Å^−3^
Δρ_min_ = −0.50 e Å^−3^

### 

Data collection: *SMART* (Bruker, 2000 ▶); cell refinement: *SAINT* (Bruker, 2000 ▶); data reduction: *SAINT*; program(s) used to solve structure: *SHELXS97* (Sheldrick, 2008 ▶); program(s) used to refine structure: *SHELXL97* (Sheldrick, 2008 ▶); molecular graphics: *PLATON* (Spek, 2009 ▶); software used to prepare material for publication: *SHELXL97*.

## Supplementary Material

Crystal structure: contains datablocks global, I. DOI: 10.1107/S1600536809007703/pk2156sup1.cif

Structure factors: contains datablocks I. DOI: 10.1107/S1600536809007703/pk2156Isup2.hkl

Additional supplementary materials:  crystallographic information; 3D view; checkCIF report

## Figures and Tables

**Table 1:** Hydrogen-bond geometry (Å, °)

*D*—H⋯*A*	*D*—H	H⋯*A*	*D*⋯*A*	*D*—H⋯*A*
C1—H1⋯Cl2	0.93	2.73	3.320 (8)	122
C10—H10⋯Cl1	0.93	2.66	3.240 (7)	121

**Fig. 1.**
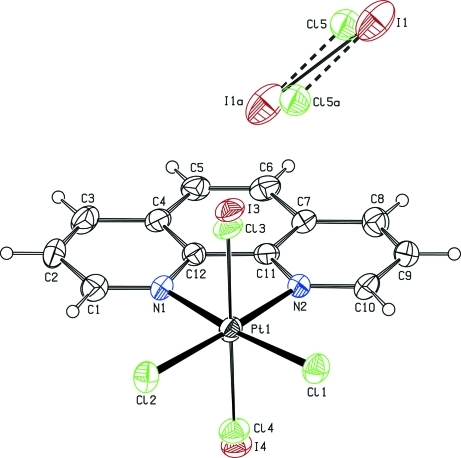
The disordered structure of the title compound, with displacement ellipsoids drawn at the 30% probability level for non-H atoms [Symmetry code: (a) 1 - x, 1 - y, -z]. The minor bonds are drawn with dashed lines.

**Fig. 2.**
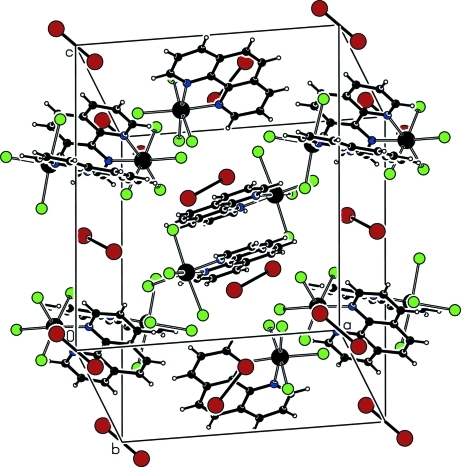
View of a packing detail of the title compound. For the sake of clarity, only the major disorder component is shown.

## supplementary crystallographic information

### Comment

The asymmetric unit of the title compound contains a neutral Pt^IV^ complex and one half-molecule of iodine which includes some Cl atoms (*ca* 6%). The Pt^IV^ ion is six-coordinated in a distorted octahedral environment by two N atoms of the 1,10-phenanthroline ligand and Cl or I atoms. The chemical formula of the pure state of the title compound would have been [PtCl_4_(C_12_H_8_N_2_)].0.5I_2_. In the particular crystal of the compound used, two Cl atoms (Cl3 and Cl4) are partially (*ca* 25% and 9%, respectively) displaced by the I atoms (I3 and I4) through the substitution reaction between the Cl^-^ and I^-^ ligand, and the I_2_ molecule also appears to have a minor component, that is I—Cl (Fig. 1 and 2). The chemical formula which resulted from the refinement of the structure was [PtCl_3.66_I_0.34_(C_12_H_8_N_2_)].0.5(Cl_0.13_I_1.87_), and in this case the ratio of the Cl atom to I atom is 2.91:1. An EDX analysis of the compound, however, gave a ratio of Cl:I = 2.47:1. Accordingly, the exact composition may very well be variable, and likely dependent on the exact conditions present during crystal formation. Even though these data are slightly different, they indicate clearly that the crystals are a solid solution in which there was some substitution of Cl for I and *vice versa*.

As a result of the different *trans* effects of the N and Cl atoms, the Pt—Cl bonds *trans* to the N atom are in general slightly shorter than bond lengths to mutually *trans* Cl atoms (Kim *et al.* 2009*a* and 2009*b*). But the *trans* effects of the N and Cl/I atoms in the crystal are not distinct owing to the disordered atoms. The Pt—I distance is restrained to the value given in table 9.6.3.3 of the International Tables Vol. C (Orpen *et al.*, 1989) (2.658 Å). The main contributor to the distortion from a true octahedral structure is the tight N1—Pt1—N2 chelate angle (81.3 (2)°), which result in non-linear *trans* axes (<Cl1—Pt1—N1 = 174.14 (16)° and <Cl2—Pt1—N2 = 175.97 (17)°). The complex displays intermolecular π-π interactions between the six-membered rings, with a shortest centroid-centroid distance of 3.771 (4) Å and with a dihedral angle between the ring planes of 2.1 (3)°. There are also weak intramolecular C—H···Cl hydrogen bonds (Table 1).

The iodine molecule was presumedly formed as a consequence of the oxidation of the iodide ion by the Pt^4+^ ion, and crystallized with the partially substituted complex. The bond distance between the I atoms is 2.708 (2) Å.

### Experimental

To a solution of [PtCl_4_(C_12_H_8_N_2_)].H_2_O (0.0821 g, 0.153 mmol) in H_2_O (20 ml) was added KI (0.1318 g, 0.794 mmol), and stirred for 2 h at room temperature. The precipitate was separated by filtration and washed with water (20 ml) and MeOH (20 ml) and dried under vacuum, to give a dark brown powder (0.0846 g). Black crystals suitable for X-ray analysis were isolated from an acetone solution of the reaction products. EDX analysis (%atom): Cl 62.30%, I 25.18%, Pt 12.52%.

### Refinement

H atoms were positioned geometrically and allowed to ride on their respective parent atoms [C—H = 0.93 Å and *U*_iso_(H) = 1.2*U*_eq_(C)]. The disordered Cl5 atom was refined isotropically. Eight restraints instructions were used for the refinement using the following *SHELXL97* (Sheldrick, 2008) commands: EADP Cl3 I3 and Cl4 I4, SIMU 0.010 I1 Cl5, BIND I1 Cl5a and Cl5 I1a, FREE Cl5 Cl5a, *DFIX* 2.658 0.010 Pt1 I3 and Pt1 I4.

### Crystal data

**Table:**

[PtCl_3.66_I_0.34_(C_12_H_8_N_2_)]·0.5(Cl_0.13_I_1.87_)	*F*(000) = 2424
*M**_r_* = 669.26	*D*_x_ = 2.795 Mg m^−^^3^
Orthorhombic, *P**b**c**a*	Mo *K*α radiation, λ = 0.71073 Å
Hall symbol: -P 2ac 2ab	Cell parameters from 973 reflections
*a* = 14.215 (5) Å	θ = 2.4–24.5°
*b* = 12.733 (5) Å	µ = 11.92 mm^−^^1^
*c* = 17.575 (6) Å	*T* = 293 K
*V* = 3180.8 (19) Å^3^	Plate, black
*Z* = 8	0.25 × 0.17 × 0.15 mm

### Data collection

**Table:**

Bruker SMART 1000 CCD diffractometer	3246 independent reflections
Radiation source: fine-focus sealed tube	2169 reflections with *I* > 2σ(*I*)
graphite	*R*_int_ = 0.049
φ and ω scans	θ_max_ = 26.4°, θ_min_ = 2.3°
Absorption correction: multi-scan (*SADABS*; Bruker, 2000)	*h* = −15→17
*T*_min_ = 0.111, *T*_max_ = 0.168	*k* = −10→15
17485 measured reflections	*l* = −17→21

### Refinement

**Table:**

Refinement on *F*^2^	Primary atom site location: structure-invariant direct methods
Least-squares matrix: full	Secondary atom site location: difference Fourier map
*R*[*F*^2^ > 2σ(*F*^2^)] = 0.031	Hydrogen site location: inferred from neighbouring sites
*wR*(*F*^2^) = 0.062	H-atom parameters constrained
*S* = 0.86	*w* = 1/[σ^2^(*F*_o_^2^) + (0.0244*P*)^2^] where *P* = (*F*_o_^2^ + 2*F*_c_^2^)/3
3246 reflections	(Δ/σ)_max_ = 0.001
194 parameters	Δρ_max_ = 1.29 e Å^−^^3^
8 restraints	Δρ_min_ = −0.50 e Å^−^^3^

### Special details

**Table:**

Geometry. All e.s.d.'s (except the e.s.d. in the dihedral angle between two l.s. planes) are estimated using the full covariance matrix. The cell e.s.d.'s are taken into account individually in the estimation of e.s.d.'s in distances, angles and torsion angles; correlations between e.s.d.'s in cell parameters are only used when they are defined by crystal symmetry. An approximate (isotropic) treatment of cell e.s.d.'s is used for estimating e.s.d.'s involving l.s. planes.
Refinement. Refinement of *F*^2^ against ALL reflections. The weighted *R*-factor *wR* and goodness of fit *S* are based on *F*^2^, conventional *R*-factors *R* are based on *F*, with *F* set to zero for negative *F*^2^. The threshold expression of *F*^2^ > 2σ(*F*^2^) is used only for calculating *R*-factors(gt) *etc*. and is not relevant to the choice of reflections for refinement. *R*-factors based on *F*^2^ are statistically about twice as large as those based on *F*, and *R*- factors based on ALL data will be even larger.

### Fractional atomic coordinates and isotropic or equivalent isotropic displacement parameters (Å^2^)

**Table:**

	*x*	*y*	*z*	*U*_iso_*/*U*_eq_	Occ. (<1)
Pt1	−0.134096 (18)	0.30394 (2)	0.177609 (15)	0.03991 (10)	
Cl1	−0.17047 (13)	0.14177 (15)	0.22685 (10)	0.0521 (5)	
Cl2	−0.28869 (12)	0.36077 (15)	0.19550 (10)	0.0512 (5)	
Cl3	−0.0903 (14)	0.3607 (17)	0.2949 (8)	0.0494 (13)	0.746 (3)
I3	−0.0759 (11)	0.3695 (13)	0.3094 (6)	0.0494 (13)	0.254 (3)
Cl4	−0.1730 (7)	0.2478 (8)	0.0572 (3)	0.0518 (8)	0.913 (3)
I4	−0.177 (2)	0.238 (2)	0.0398 (9)	0.0518 (8)	0.087 (3)
N1	−0.0875 (4)	0.4446 (4)	0.1355 (3)	0.0393 (14)	
N2	0.0029 (4)	0.2642 (4)	0.1585 (3)	0.0388 (13)	
C1	−0.1341 (5)	0.5328 (6)	0.1283 (4)	0.0517 (19)	
H1	−0.1978	0.5341	0.1403	0.062*	
C2	−0.0911 (6)	0.6243 (6)	0.1031 (4)	0.058 (2)	
H2	−0.1256	0.6863	0.0999	0.070*	
C3	0.0015 (6)	0.6235 (6)	0.0831 (4)	0.055 (2)	
H3	0.0305	0.6846	0.0660	0.066*	
C4	0.0535 (5)	0.5285 (6)	0.0887 (4)	0.0438 (18)	
C5	0.1496 (5)	0.5174 (6)	0.0688 (4)	0.0484 (19)	
H5	0.1820	0.5741	0.0481	0.058*	
C6	0.1946 (5)	0.4255 (6)	0.0796 (4)	0.050 (2)	
H6	0.2581	0.4210	0.0672	0.061*	
C7	0.1485 (5)	0.3347 (5)	0.1093 (4)	0.0403 (17)	
C8	0.1899 (5)	0.2382 (6)	0.1246 (4)	0.052 (2)	
H8	0.2534	0.2282	0.1141	0.062*	
C9	0.1393 (5)	0.1590 (6)	0.1546 (4)	0.0494 (19)	
H9	0.1678	0.0947	0.1647	0.059*	
C10	0.0446 (5)	0.1735 (5)	0.1703 (4)	0.0447 (18)	
H10	0.0098	0.1177	0.1897	0.054*	
C11	0.0538 (5)	0.3460 (6)	0.1273 (4)	0.0384 (17)	
C12	0.0055 (5)	0.4415 (5)	0.1167 (3)	0.0368 (16)	
I1	0.56522 (7)	0.45323 (7)	0.04476 (5)	0.0912 (4)	0.936 (3)
Cl5	0.558 (3)	0.496 (4)	0.021 (2)	0.062 (9)*	0.064 (3)

### Atomic displacement parameters (Å^2^)

**Table:**

	*U*^11^	*U*^22^	*U*^33^	*U*^12^	*U*^13^	*U*^23^
Pt1	0.04138 (16)	0.03167 (16)	0.04668 (17)	−0.00138 (13)	0.00129 (14)	0.00057 (14)
Cl1	0.0561 (11)	0.0392 (11)	0.0608 (12)	−0.0060 (9)	0.0047 (9)	0.0072 (9)
Cl2	0.0490 (11)	0.0431 (12)	0.0616 (12)	0.0050 (9)	0.0083 (9)	0.0040 (9)
Cl3	0.064 (4)	0.045 (3)	0.039 (4)	−0.005 (2)	−0.007 (2)	−0.014 (3)
I3	0.064 (4)	0.045 (3)	0.039 (4)	−0.005 (2)	−0.007 (2)	−0.014 (3)
Cl4	0.0649 (15)	0.052 (2)	0.038 (3)	−0.0073 (14)	−0.006 (3)	−0.004 (2)
I4	0.0649 (15)	0.052 (2)	0.038 (3)	−0.0073 (14)	−0.006 (3)	−0.004 (2)
N1	0.044 (4)	0.029 (4)	0.045 (3)	−0.001 (3)	−0.001 (3)	−0.002 (3)
N2	0.037 (3)	0.032 (3)	0.046 (3)	0.000 (3)	−0.005 (3)	−0.001 (3)
C1	0.053 (5)	0.048 (5)	0.055 (5)	0.001 (4)	−0.002 (4)	−0.007 (4)
C2	0.057 (5)	0.031 (5)	0.086 (6)	0.006 (4)	−0.009 (5)	−0.004 (4)
C3	0.071 (6)	0.034 (5)	0.060 (5)	−0.011 (4)	−0.003 (4)	0.003 (4)
C4	0.052 (5)	0.036 (5)	0.043 (4)	−0.006 (4)	−0.007 (4)	−0.003 (3)
C5	0.050 (5)	0.044 (5)	0.051 (5)	−0.017 (4)	−0.006 (4)	0.007 (4)
C6	0.045 (5)	0.052 (6)	0.054 (5)	−0.012 (4)	0.006 (4)	−0.011 (4)
C7	0.044 (5)	0.036 (4)	0.041 (4)	−0.001 (3)	−0.002 (3)	−0.006 (3)
C8	0.046 (5)	0.053 (5)	0.056 (5)	0.007 (4)	0.006 (4)	−0.004 (4)
C9	0.047 (5)	0.046 (5)	0.055 (5)	0.009 (4)	−0.007 (4)	−0.010 (4)
C10	0.050 (5)	0.032 (5)	0.052 (5)	−0.006 (3)	0.002 (4)	−0.002 (3)
C11	0.040 (4)	0.037 (4)	0.038 (4)	−0.007 (3)	−0.008 (3)	−0.001 (3)
C12	0.042 (4)	0.034 (4)	0.034 (4)	−0.002 (3)	−0.004 (3)	0.002 (3)
I1	0.1295 (7)	0.0635 (6)	0.0805 (6)	−0.0192 (5)	0.0312 (5)	−0.0058 (4)

### Geometric parameters (Å, °)

**Table:**

Pt1—N2	2.040 (5)	C4—C12	1.391 (9)
Pt1—N1	2.048 (5)	C4—C5	1.417 (9)
Pt1—Cl3	2.272 (9)	C5—C6	1.346 (9)
Pt1—Cl1	2.2977 (19)	C5—H5	0.9300
Pt1—Cl4	2.301 (4)	C6—C7	1.428 (9)
Pt1—Cl2	2.3347 (19)	C6—H6	0.9300
Pt1—I3	2.598 (7)	C7—C8	1.389 (9)
Pt1—I4	2.635 (9)	C7—C11	1.391 (8)
N1—C1	1.309 (8)	C8—C9	1.346 (10)
N1—C12	1.364 (8)	C8—H8	0.9300
N2—C10	1.316 (8)	C9—C10	1.387 (9)
N2—C11	1.381 (8)	C9—H9	0.9300
C1—C2	1.388 (10)	C10—H10	0.9300
C1—H1	0.9300	C11—C12	1.408 (9)
C2—C3	1.364 (10)	I1—Cl5^i^	2.19 (4)
C2—H2	0.9300	I1—I1^i^	2.708 (2)
C3—C4	1.421 (10)	Cl5—I1^i^	2.19 (4)
C3—H3	0.9300		
			
N2—Pt1—N1	81.3 (2)	N1—C1—H1	119.0
N2—Pt1—Cl3	88.1 (6)	C2—C1—H1	119.0
N1—Pt1—Cl3	87.8 (6)	C3—C2—C1	120.0 (7)
N2—Pt1—Cl1	93.11 (17)	C3—C2—H2	120.0
N1—Pt1—Cl1	174.14 (16)	C1—C2—H2	120.0
Cl3—Pt1—Cl1	90.3 (6)	C2—C3—C4	119.4 (7)
N2—Pt1—Cl4	90.0 (3)	C2—C3—H3	120.3
N1—Pt1—Cl4	91.0 (3)	C4—C3—H3	120.3
Cl3—Pt1—Cl4	177.9 (6)	C12—C4—C5	118.7 (7)
Cl1—Pt1—Cl4	90.8 (3)	C12—C4—C3	116.6 (7)
N2—Pt1—Cl2	175.97 (17)	C5—C4—C3	124.7 (7)
N1—Pt1—Cl2	94.69 (16)	C6—C5—C4	120.7 (7)
Cl3—Pt1—Cl2	92.1 (5)	C6—C5—H5	119.7
Cl1—Pt1—Cl2	90.91 (7)	C4—C5—H5	119.7
Cl4—Pt1—Cl2	89.7 (3)	C5—C6—C7	122.5 (7)
N2—Pt1—I3	85.6 (4)	C5—C6—H6	118.8
N1—Pt1—I3	86.5 (4)	C7—C6—H6	118.8
Cl3—Pt1—I3	2.7 (9)	C8—C7—C11	117.2 (7)
Cl1—Pt1—I3	91.4 (4)	C8—C7—C6	126.4 (7)
Cl4—Pt1—I3	175.2 (4)	C11—C7—C6	116.3 (6)
Cl2—Pt1—I3	94.6 (4)	C9—C8—C7	120.8 (7)
N2—Pt1—I4	89.4 (8)	C9—C8—H8	119.6
N1—Pt1—I4	91.3 (7)	C7—C8—H8	119.6
Cl3—Pt1—I4	177.5 (9)	C8—C9—C10	119.8 (7)
Cl1—Pt1—I4	90.4 (7)	C8—C9—H9	120.1
Cl4—Pt1—I4	0.7 (10)	C10—C9—H9	120.1
Cl2—Pt1—I4	90.3 (7)	N2—C10—C9	121.5 (7)
I3—Pt1—I4	174.8 (8)	N2—C10—H10	119.2
C1—N1—C12	119.5 (6)	C9—C10—H10	119.2
C1—N1—Pt1	128.4 (5)	N2—C11—C7	121.3 (6)
C12—N1—Pt1	112.1 (4)	N2—C11—C12	116.6 (6)
C10—N2—C11	119.3 (6)	C7—C11—C12	122.0 (6)
C10—N2—Pt1	128.5 (5)	N1—C12—C4	122.5 (6)
C11—N2—Pt1	112.2 (4)	N1—C12—C11	117.7 (6)
N1—C1—C2	121.9 (7)	C4—C12—C11	119.7 (6)
			
N2—Pt1—N1—C1	−176.8 (6)	C3—C4—C5—C6	176.6 (7)
Cl3—Pt1—N1—C1	−88.4 (8)	C4—C5—C6—C7	1.6 (11)
Cl4—Pt1—N1—C1	93.3 (6)	C5—C6—C7—C8	−177.6 (7)
Cl2—Pt1—N1—C1	3.6 (6)	C5—C6—C7—C11	−0.4 (10)
I3—Pt1—N1—C1	−90.8 (7)	C11—C7—C8—C9	1.1 (10)
I4—Pt1—N1—C1	93.9 (9)	C6—C7—C8—C9	178.3 (7)
N2—Pt1—N1—C12	0.2 (4)	C7—C8—C9—C10	0.1 (11)
Cl3—Pt1—N1—C12	88.7 (7)	C11—N2—C10—C9	2.1 (9)
Cl4—Pt1—N1—C12	−89.7 (5)	Pt1—N2—C10—C9	−179.7 (5)
Cl2—Pt1—N1—C12	−179.4 (4)	C8—C9—C10—N2	−1.7 (10)
I3—Pt1—N1—C12	86.3 (5)	C10—N2—C11—C7	−0.9 (9)
I4—Pt1—N1—C12	−89.0 (8)	Pt1—N2—C11—C7	−179.4 (5)
N1—Pt1—N2—C10	−179.6 (6)	C10—N2—C11—C12	−179.4 (6)
Cl3—Pt1—N2—C10	92.4 (8)	Pt1—N2—C11—C12	2.1 (7)
Cl1—Pt1—N2—C10	2.2 (5)	C8—C7—C11—N2	−0.7 (9)
Cl4—Pt1—N2—C10	−88.6 (6)	C6—C7—C11—N2	−178.2 (6)
I3—Pt1—N2—C10	93.4 (7)	C8—C7—C11—C12	177.7 (6)
I4—Pt1—N2—C10	−88.2 (9)	C6—C7—C11—C12	0.3 (9)
N1—Pt1—N2—C11	−1.3 (4)	C1—N1—C12—C4	−1.0 (9)
Cl3—Pt1—N2—C11	−89.3 (7)	Pt1—N1—C12—C4	−178.3 (5)
Cl1—Pt1—N2—C11	−179.5 (4)	C1—N1—C12—C11	178.2 (6)
Cl4—Pt1—N2—C11	89.7 (5)	Pt1—N1—C12—C11	0.9 (7)
I3—Pt1—N2—C11	−88.3 (6)	C5—C4—C12—N1	−178.4 (6)
I4—Pt1—N2—C11	90.1 (8)	C3—C4—C12—N1	2.3 (9)
C12—N1—C1—C2	−1.2 (10)	C5—C4—C12—C11	2.4 (9)
Pt1—N1—C1—C2	175.7 (5)	C3—C4—C12—C11	−176.9 (6)
N1—C1—C2—C3	1.8 (11)	N2—C11—C12—N1	−2.0 (9)
C1—C2—C3—C4	−0.4 (11)	C7—C11—C12—N1	179.5 (6)
C2—C3—C4—C12	−1.6 (10)	N2—C11—C12—C4	177.2 (6)
C2—C3—C4—C5	179.2 (7)	C7—C11—C12—C4	−1.3 (10)
C12—C4—C5—C6	−2.6 (10)		

Symmetry codes: (i) −*x*+1, −*y*+1, −*z*.

### Hydrogen-bond geometry (Å, °)

**Table:**

*D*—H···*A*	*D*—H	H···*A*	*D*···*A*	*D*—H···*A*
C1—H1···Cl2	0.93	2.73	3.320 (8)	122
C10—H10···Cl1	0.93	2.66	3.240 (7)	121
